# The Utility of Lymphatic Massage in Cosmetic Procedures

**DOI:** 10.1093/asjof/ojad023

**Published:** 2023-02-28

**Authors:** Troy Marxen, Orr Shauly, Pedram Goel, Tina Tsan, Rebecca Faria, Daniel J Gould

## Abstract

Lymphedema is a severe debilitating disease characterized by the accumulation of excessive protein-rich fluid in the interstitial space. Given the severe morbidity associated with this disease process, various surgical and nonsurgical treatment modalities have been developed to attempt to reduce the incidence and symptoms associated with lymphedema. Manual lymphatic drainage (MLD) is a component of complete decongestive therapy on-surgical treatment which has demonstrated benefit in reducing the development of lymphedema following surgery. Here we provide a review of literature on MLD and its potential mechanism of action. This paper aims to educate patients, physicians, and surgeons about MLD regarding its efficacy and utility in the treatment paradigm for lymphedema and to translate concepts from the treatment of lymphedema to cosmetic procedures.

**Level of Evidence: 5:**

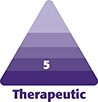

Lymphedema is a disease process manifested by inadequate drainage of lymphatic vessels, resulting in an accumulation of excess protein-rich interstitial fluid. Under homeostatic conditions, fluid interactions between capillaries, interstitial space, and lymphatic vessels allow for net fluid to extravasate from the arterial end of capillaries, which is then reabsorbed into lymphatic channels at the venous side of capillaries. The delicate balance of hydrostatic pressure and colloid pressure between the capillaries and interstitial spaces normally work in harmony to prevent the accumulation and stasis of fluid. Under normal circumstances, any excess fluid in the interstitial space is easily and readily removed by a nonpathologic lymphatic system. Upon entering the lymphatic vessels, the fluid, now termed lymph, circulates through the lymphatic vessels before ultimately ending up returning into the venous circulation.^1^ Any disruption to this delicate lymphatic system and its equilibrium can result in the development of severe and debilitating lymphedema. Notably this equilibrium is routinely disrupted by surgical procedures, and cosmetic procedures including liposuction, facial surgery, and body contouring.

Lymphedema has traditionally been classified based on etiology, broadly divided into inherited (primary) or acquired (secondary) forms. Primary causes of lymphedema are rare, when compared with the acquired forms, and often a result of genetic abnormalities.^2,3^ The majority of lymphedema cases encountered in health care are therefore acquired, occurring secondary to accidental or iatrogenic trauma or other lymphatic-disrupting disease processes. Plastic surgeons are generally familiar with the most common causes of secondary lymphedema which include cancer, chemotherapy, radiation, lymph-node dissection, and various surgical procedures. Additionally, complications that stem from these secondary causes of lymphedema, such as infection and seroma, can further exacerbate or result in lymphedema.

Given the prevalence of secondary causes of lymphedema, many treatment modalities have been developed to help reduce the risk of developing lymphedema or provide symptom relief. Treatments span from nonsurgical complete decongestive therapy (CDT) and massage to surgical interventions which include lymphaticovenous bypass, vascularized lymph node transfer, laser treatment, and stem-cell therapy.^4–6^ CDT is now widely accepted as the mainstay conservative treatment approach for lymphedema and consists of a multi-factorial approach which include skin care, exercise, compression garments, and manual lymphatic drainage (MLD).^7,8^ This MLD is a massage technique that is thought to decrease fibrosis and lymphatic rerouting to functioning lymphatic channels and serves as the most common modality used to assist in lymphatic restoration following potential lymphatic disruption during surgery.^1,9–11^

## LYMPHATIC MASSAGE: PROPOSED MECHANISM IN IMPROVING LYMPHATIC DRAINAGE

MLD utilizes gentle massaging along lymphatic channels with slow repetitive movements to reduce buildup of fluid in extremities following surgery; however, the exact physiologic mechanism by which MLD exerts its effect remains unclear.^10,12^ Multiple studies have attempted to uncover its specific mechanism of action; however, MLD physiology remains poorly understood with various conflicting hypothesis proposed in the literature. Some have proposed that by performing MLD, lymphatic vessels respond by increasing their transport ability through increased contractions.^13,14^ Others believe that MLD increases interstitial pressure which results in improved lymphatic reabsorption.^15,16^ Further hypotheses propose that MLD promotes a decrease in distal lymphatic pressure, enhancing uptake, or that MLD improves accessory lymphatic routes that enhance lymphatic reuptake.^17,18^ There is evidence that this effect may be multifactorial with one study demonstrating reduced limb volume following MLD despite no change in lymph transport.^19^

Lymphatic function effects have been a primary source of investigation for patients undergoing MLD, but data suggest there may also be beneficial effects on arteriovenous system which in turn helps to reduce fluid accumulation. One possible mechanism by which MLD reduces microlymphatic hypertension is by increasing peripheral arterial blood flow through superficial circulation or skin circulation.^13,20^ One study utilizing a mouse model demonstrated increased femoral vein blood flow as a result of MLD,^17^ suggesting this method can help to improve systemic venous return. Other studies have suggested that the mechanism of improvement in lymphatic drainage may even be further upstream, with effects on the autonomic nervous system.^21^ Various studies have demonstrated improved breathlessness, sleep, and increased urinary secretion of adrenergic hormones, serotonin, and histamine as a result of MLD.^22,23^

## LYMPHATIC MASSAGE VARIATIONS

There are 4 main methods of MLD, which are used in conjunction for the treatment of lymphedema along with exercise, compression therapy, and proper skin-care treatments. In all the 4 most common techniques of MLD, hand movements are strategically placed in areas to apply tension on the skin to subsequently increase interstitial pressures, theoretically promoting lymphatic uptake. Movements are rhythmic, at a controlled pace, and allow for a return phase in which the skin under tension can return to normal tone. Applied pressure can vary and may depend on the tissue, with more severe lymphedema requiring greater pressure if there are fibrotic or sclerotic areas, deeper pressure may be necessary.

Although seemingly counterintuitive, MLD usually begins with treatment of the central or proximal region and works distally, in contrast to pressure garments that depend on a distal to proximal restoration of flow. Additionally, attention may be given to the contralateral (healthy) side to promote better lymphatic drainage at a systemic level.^12^ All MLD techniques may incorporate training on proper breathing to reduce intrathoracic and intraabdominal pressure to theoretically enhance deeper lymphatic uptake.^22^

There are various nuances to the different MLD techniques. One of the first treatment methods was developed in the 1930s by Emil Vodder, now coined the “Vodder method.” This method includes different hand movements, emphasizing circular movements of the thumb, including the “thumb circle,” “stationary circle,” “pump,” “scoop,” and “rotary” movements.^24^ These specialized hand movements are used to direct fluid from one quadrant of the body across an anastomoses—a connection between the body's fluid drainage pathways. This is how a therapist moves fluid from a region of the body where the lymphatics are impaired to another region of the body where the lymphatics are functioning. A trained therapist will locate inter-territorial anastomosis and help re-route the lymph around impaired lymph nodes or absent lymph vessels, toward healthy ones. The Földi technique was developed as an offshoot of the Vodder technique and utilizes similar hand movements described previously, with the addition of a period of increased pressure followed by a relaxation phase. The Földi technique also incorporates the “encircling” stoke to help reduce edema.^25^ The Casley–Smith method added the technique consisting of using the side of the hand over specific watershed areas between distinct skin lymph territories with a slow and gentle “efflurage” maneuver.^15^ Leduc added “call-up” and “reabsorption” maneuvers to sequentially promote lymphatic uptake in the distal to proximal regions of lymphatic uptake.^16^ Any of the 4 most common MLD techniques may be suitable for treating a patient with a lymphedema, and careful selection of the technique may depend on the patient and method in which the therapist is best trained.

## DISCUSSION

**Figure 1.**
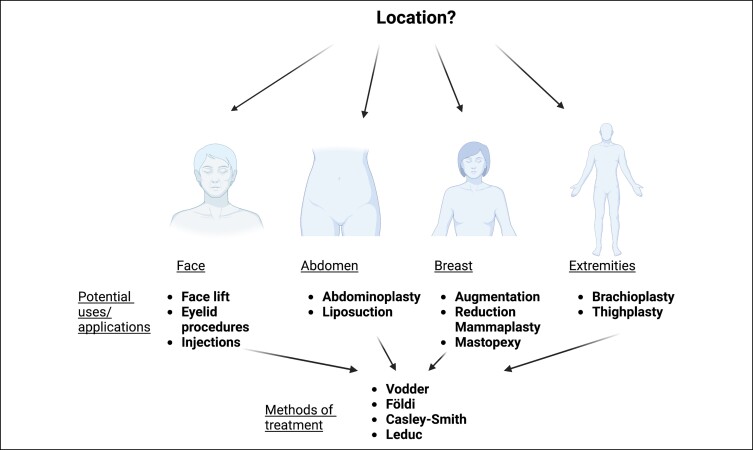
Potential uses and applications of lymphatic massage in plastic and reconstructive surgery.

There are many potential use cases for MLD in plastic surgery patients (Figure 1). Originally, MLD had been utilized in postmastectomy breast-cancer patients prior to undergoing reconstruction, with notable improvements in pain and swelling.^10,22^ Recent studies have begun to expand the use of MLD to those who have undergone cosmetic procedures. In the case of cosmetic plastic surgery, the postoperative period is one of the most critical phases. General postoperative manual lymphatic massage recommendations consist of manual lymphatic massage 2 to 3 times per week during the initial 3 to 4 weeks of recovery to be performed by a certified lymphedema therapist or a licensed massage therapist who have undergone some degree of lymphedema training and are often certified to perform postoperative lymphatic drainage techniques. It is through postoperative care that the expected results are achieved. Rapidly reducing lymphostasis postoperatively may help improve early results and prevent late sequelae. In addition to fighting this accumulation of fluid, doing lymphatic drainage after plastic surgery may help in recovery, improving blood circulation, reducing pain, and facilitating healing. Fibrosis, an inflammatory process that causes hardening of the skin, is a common concern for those recovering from plastic surgery. Aesthetic procedures, especially liposuction, results in postoperative swelling that can take between 3 and 6 months for the body's lymphatic system to resolve. Similar to skin pathophysiology of chronic lymphedema, fluid accumulation after liposuction can create hardened lumps and bumps of the skin. However, unlike lymphedema, these lumps and bumps can be fully resolved with MLD and compression garments; patients undergoing elective cosmetic procedures have healthy intact lymphatic systems, while lymphedema patients do not. Since the recovery process from plastic surgery can be a lengthy one, between 3 and 6 months, utilizing MLD therapy during the postoperative recovery phase, can be beneficial in supporting the lymphatic system in its role in reabsorption of fluid.

### Abdomen

In patients undergoing abdominal liposuction, the superficial lymphatic system is at an elevated risk of disruption due to large, sweeping cannula strokes, often resulting in lymphatic stasis.^26^ In abdominoplasty, it has been demonstrated that the primary source of lymphatic drainage may shift from inguinal to axillary after undergoing surgery, also placing one at an elevated risk for stasis and potential edema.^27^ Lymphedema stemming from cosmetic procedures presents a significant burden to patients who are seeking improved aesthetics by means of often elective procedures, as it can negatively affect comfort, quality of life, and activities of daily living.^28^ Numerous studies have found that MLD in conjunction with therapeutic adjuncts can provide reductions in edema, fibrosis, as well as provided analgesia in patients undergoing liposuction and/or lipoabdominoplasty.^28–31^ These been found to extended from the abdomen to all core liposuction areas, including the upper and lower abdomen, flanks, back, and hips.^31^

### Breast

In contrast to mastectomy and reconstruction, there are fewer known benefits to performing MLD in patients undergoing primary cosmetic procedures. Some studies have incorporated MLD in their postoperative protocol for patients undergoing breast augmentation.^32–34^ Additionally, multiple surgeons performing mastopexy have incorporated MLD into their postoperative protocols.^35,36^ There are few studies evaluating the use of MLD in reduction mammaplasty, but some studies have shown altered lymphatic drainage following breast procedures, including breast reduction, suggesting a potential benefit for the incorporation of MLD into postoperative management.^37,38^

### Face

MLD use is less well studied in other areas of cosmetic procedures but may have some use in mitigating complications after facial procedures such as injectables, fillers, or botulinum toxin.^39,40^ Some practices are incorporating MLD into postoperative recovery from facelift, with the goal of minimizing swelling and pain.^41–43^ Therefore, MLD may further provide benefit in improving postoperative edema in the periorbital region after eyelid procedures.^44^

### Extremity

Studies evaluating the use of MLD following cosmetic procedures are somewhat limited; however, its use is most commonly described following thigh lift or brachioplasty in the massive weight loss population. Although contemporary surgical techniques are effective in improving body contouring and skin tightness, the surgeon should be aware that excess resection can result in significant alterations in lymphatic drainage.^45^ This has led some surgeons to suggest that MLD be utilized as a useful adjunct to reduce the risk of developing postoperative lymphedema.^46,47^

## CONCLUSIONS

As the number of patients undergoing cosmetic procedures continues to increase, there is a need for adjunctive therapies that promote patient recovery which is paramount to improve postoperative outcomes and enhance patient's satisfaction and aesthetic desires. MLD is an area of active research that has preliminarily shown a benefit in patients undergoing a variety of aesthetic procedures. Due to the paucity of research on MLD in aesthetic procedures, more studies on the topic are warranted.
